# Comparison of Multiple Bioactive Constituents in the Corolla and Other Parts of *Abelmoschus manihot*

**DOI:** 10.3390/molecules26071864

**Published:** 2021-03-25

**Authors:** Shengxin Yin, Yuqi Mei, Lifang Wei, Lisi Zou, Zhichen Cai, Nan Wu, Jiahuan Yuan, Xunhong Liu, Haitao Ge, Dianguang Wang, Dandan Wang

**Affiliations:** 1 College of Pharmacy, Nanjing University of Chinese Medicine, Nanjing 210023, China; yinshengxin723@163.com (S.Y.); 18260028173@163.com (Y.M.); weilifangquiet@163.com (L.W.); zlstcm@126.com (L.Z.); caizhichen2008@126.com (Z.C.); wunan7272@163.com (N.W.); yuanjiahuan1027@163.com (J.Y.); 2 SZYY Group Pharmaceutical Limited, Taizhou 225500, China; geht@suzhongyy.com (H.G.); wangdg@suzhongyy.com (D.W.); wangdd@suzhongyy.com (D.W.)

**Keywords:** *Abelmoschus manihot*, root, stem, leaf, corolla, seed, bioactive constituents, distribution patterns

## Abstract

*Abelmoschus manihot* (L.) Medic (AM), called Huangshukui in Chinese, is a widely used medicinal plant. Each part of AM has medicinal value, including Abelmoschi Radix (AR), Abelmoschi Herba (AH), Abelmoschi Folium (AF), Abelmoschi Corolla (AC), and Abelmoschi Semen (AS). However, only AC is documented in the Chinese Pharmacopoeia. In order to investigate whether there is any difference between AC and the other parts of AM, an analytical method based on ultra-fast performance liquid chromatography coupled with triple quadrupole-linear ion trap mass spectrometry (UFLC-QTRAP-MS/MS) was established for the simultaneous determination of 35 constituents in different parts of AM. Moreover, principal components analysis (PCA) and partial least squares discriminant analysis (PLS-DA) were applied to classify and evaluate the different parts of AM based on the content of the 35 constituents. The total contents of the 35 constituents in AC were significantly higher than in the other parts of AM and the results revealed significant differences between AC and the other parts of AM. Eight constituents were remarkably related to the sample classifications. This research does not just provide the basic information for revealing the distribution patterns in different parts of AM from the same origin, but also complements some of the scientific data for the comprehensive quality evaluation of AC.

## 1. Introduction

Abelmoschi Corolla (AC), which is the dried corolla of *Abelmoschus manihot* (AM) in the Chinese Pharmacopoeia (2020 version) [1], has widespread use in the Chinese medicine industry. It is widely applied in the treatment of inflammation, primary glomerular disease and type 2 diabetic nephropathy [2,3,4] in China, Papua New Guinea, Vanuatu, Fiji and New Caledonia [5]. In addition, other parts of AM, including Abelmoschi Radix (AR), Abelmoschi Herba (AH), Abelmoschi Folium (AF), and Abelmoschi Semen (AS) have also been recorded in previous works for medicinal purposes [6]. Chemical composition is the basis of the pharmacological action of traditional Chinese medicine. Phytochemical analysis has revealed that AC contains multiple chemical constituents, such as flavonoids [7], organic acids [8], nucleosides, and amino acids [9,10]. Among these constituents, flavonoids and organic acids possess various pharmacological activities including anti-inflammatory, antioxidant, anti-tumor [11,12,13], and neuroprotective effects [14,15]; nucleosides are biologically active ingredients that enhance immunity and antiviral effects [16]; amino acids are the essential nutrients conducive to the human body, which also exhibit excellent pharmacological activity, including antioxidant and anti-hypertensive activity [17,18]. The effects of these constituents are consistent with the pharmacological effects of AC. Meanwhile, other parts of AM have similar chemicals to AC and studies have shown that AH and AF have the effect of promoting wound healing and analgesia, respectively [19,20]. However, except for AC, other parts of AM have not been used frequently while a comparative study among different parts of AM is also in a preliminary stage. Therefore, it is necessary to develop a reliable method to study the distribution patterns of metabolites in different parts of AM, with the hope of providing basic data for quality evaluation research on AC.

In the present study, a reliable and comprehensive method based on ultra-fast performance liquid chromatography coupled with triple quadrupole-linear ion trap mass spectrometry (UFLC-QTRAP-MS/MS) was established for the simultaneous determination of 35 constituents in the different parts of AM, including 14 flavonoids, eight organic acids, three nucleosides, and 10 amino acids. Furthermore, multivariate statistical analysis was applied to this study based on the content of the 35 constituents. Principal components analysis (PCA) was utilized to classify the samples [21,22]. Then partial least squares discriminant analysis (PLS-DA) was performed to find out the important metabolites that cause classification [23]. This research provides the basic information for revealing the accumulation laws of metabolites in different parts of AM from the same origin, and also complements some of the scientific data for the comprehensive quality evaluation of AC.

## 2. Results

### 2.1. Optimization of Extraction Conditions

The optimal extraction condition was 70% methanol as the extraction solvent, with a solid–liquid ratio of 1:40 g/mL, and ultrasonic extraction for 30 min using the single factor test.

### 2.2. Optimization of UFLC and Mass Spectrometric Conditions

After experimental verification analysis, chromatographic separation was performed on an XBridge^®^C_18_ column (4.6 mm × 100 mm, 3.5 μm) at 30 °C with a gradient elution of 0.1% (*v*/*v*) aqueous formic acid water solution (A)–methanol:acetonitrile (1:1) (B) at a flow rate of 0.5 mL/min. The injection volume was 2 µL and the elution gradient was optimized as follows: 0–5 min, 2–20% B; 5–13 min, 20–25% B; 13–26 min, 25–30% B; 26–28 min, 30–75% B; 28–31 min, 75–95% B.

**Figure 1:**
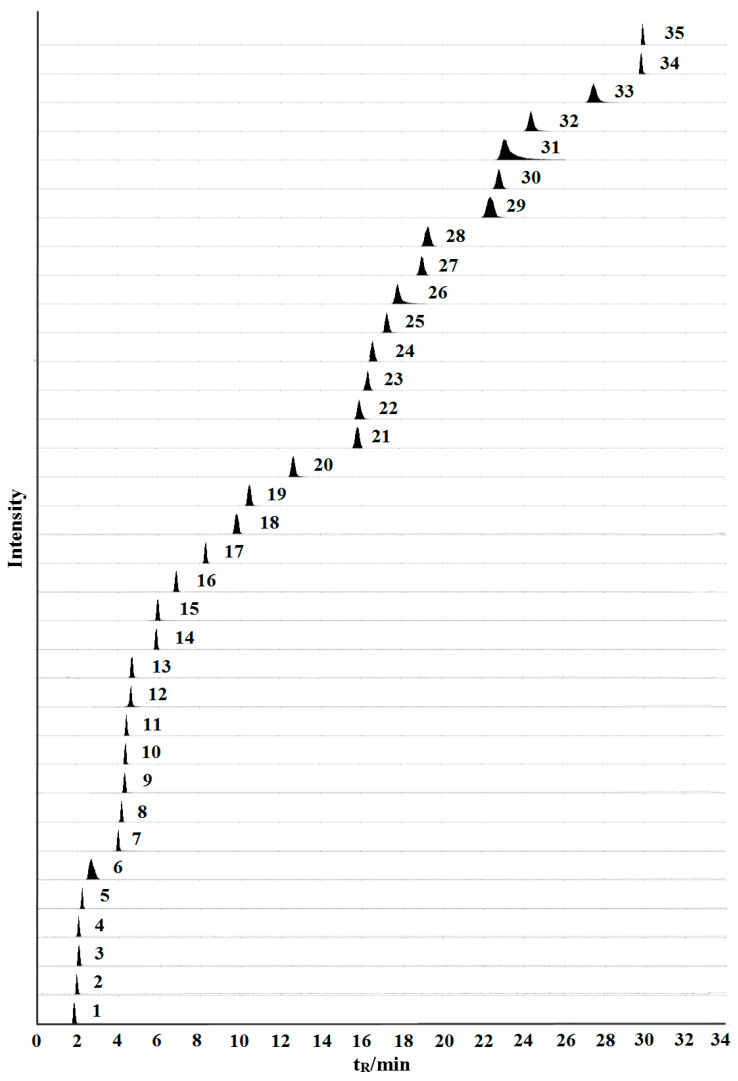
Representative extract ion chromatograms (XIC) of multiple-reaction monitoring (MRM) chromatograms of the 35 investigated constituents. (The peak numbers denoted are the same as those in Table 1).

The constituents were detected under multiple-reaction monitoring (MRM) mode. The flavonoids and organic acids were detected in negative ion mode. Amino acids and nucleosides were detected in positive ion mode. The optimized mass spectrometry parameters, including MRM transitions, as well as the declustering potential (DP) and collision energy (CE) of the 35 constituents are listed in Table 1. The MRM of the 35 constituents are shown in Figure 1.

### 2.3. Method Validation

The details of the validation results of the method are presented in Table 2. The standard calibration curves showed good linearity with appropriate correlation coefficients (r > 0.9990). The limits of detections and quantifications (LODs and LOQs) ranged from 0.07–66.00 ng/mL and 0.22–220.00 ng/mL, respectively, which indicated the high sensitivity of the method. The relative standard deviation (RSD) of intra-day and inter-day precision, repeatability, and stability of all constituents ranged from 1.1% to 4.9%, 3.6% to 4.9%, 1.0% to 4.9%, and 2.1% to 4.9%, respectively. The overall recoveries varied from 98.06% to 104.4%, with RSDs < 5.0%, indicating this method was validated for all constituents.

### 2.4. Quantitative Analysis of Samples

**Figure 2:**
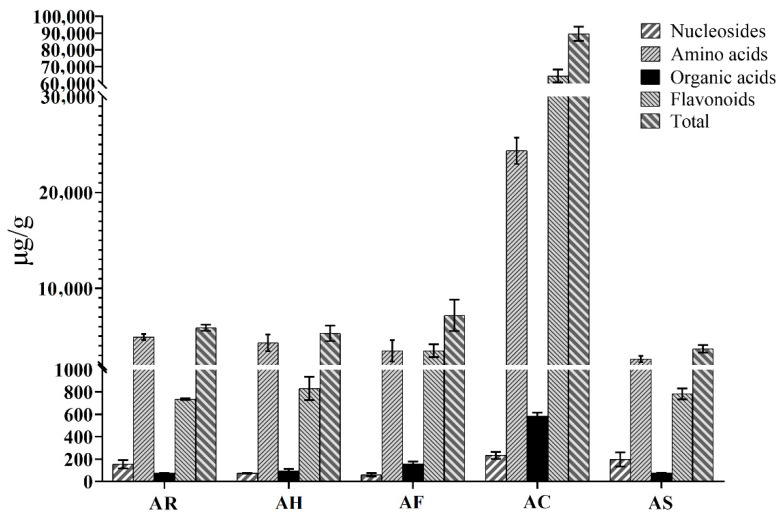
The content of four types of constituents in different parts of *Abelmoschus manihot*.

The developed UFLC-QTRAP-MS/MS method was subsequently applied to the simultaneous determination of multiple constituents in different parts of AM. The quantitative results of the 35 constituents are presented in Tables S1 and S2. As shown in Figure 2. The contents of flavonoids and amino acids account for a high proportion in the different parts of AM, and the contents of nucleosides and organic acids in each part of AM are at a relatively low level. By comparison of the chemical content in the different parts of AM, we found that AC was quite different from the others. Total contents of flavonoids in AC ranged from 60,905.55–69,851.44 µg/g, while the contents of other parts ranged from 728.30–4600.97 µg/g. Total contents of amino acids in AC ranged from 23,114.76–26,704.54 µg/g, while the contents of other parts ranged from 2223.47–5814.70 µg/g. The flavonoids and amino acids in AM were mainly distributed in AC. The ranges of nucleosides were 204.32–277.00 µg/g in AC and 41.07–279.95 µg/g in other parts of AM. The ranges of organic acids were 554.90–624.45 µg/g in AC and 71.00–178.19 µg/g in other parts of AM, respectively. This result proved that the proportion of nucleosides and organic acids in each part was relatively low.

### 2.5. Distribution of Bioactive Constituents among AC and Other Parts of AM

**Figure 3:**
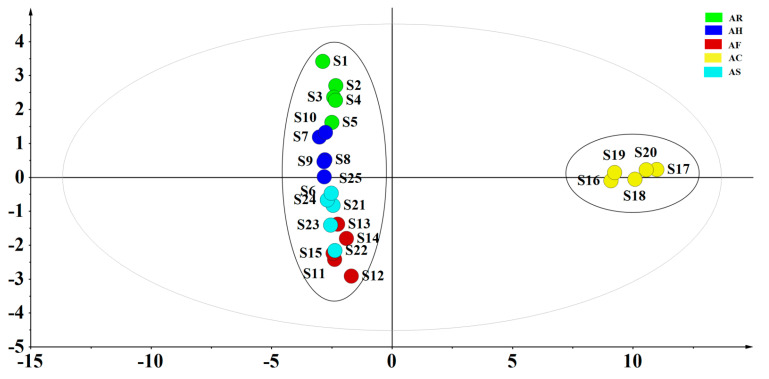
The principal component analysis (PCA) scores scatter plot of different parts of *Abelmoschus manihot*.

PCA was performed to classify and distinguish different parts of AM according to the contents of the 35 constituents. The first two principal components accounted for more than 80%, which could be used to represent the overall information of the samples (R^2^X [1] = 0.748, R^2^X [2] = 0.0817). As shown in Figure 3. The PCA scores plot indicated that AC and other parts of AM were divided into two clusters. Samples of AC were gathered in the positive axis of t [1], while AR, AH, AF, and AS were distributed in the negative axis of t [1]. It is obvious that there were significant differences between AC and other parts of AM.

**Figure 4:**
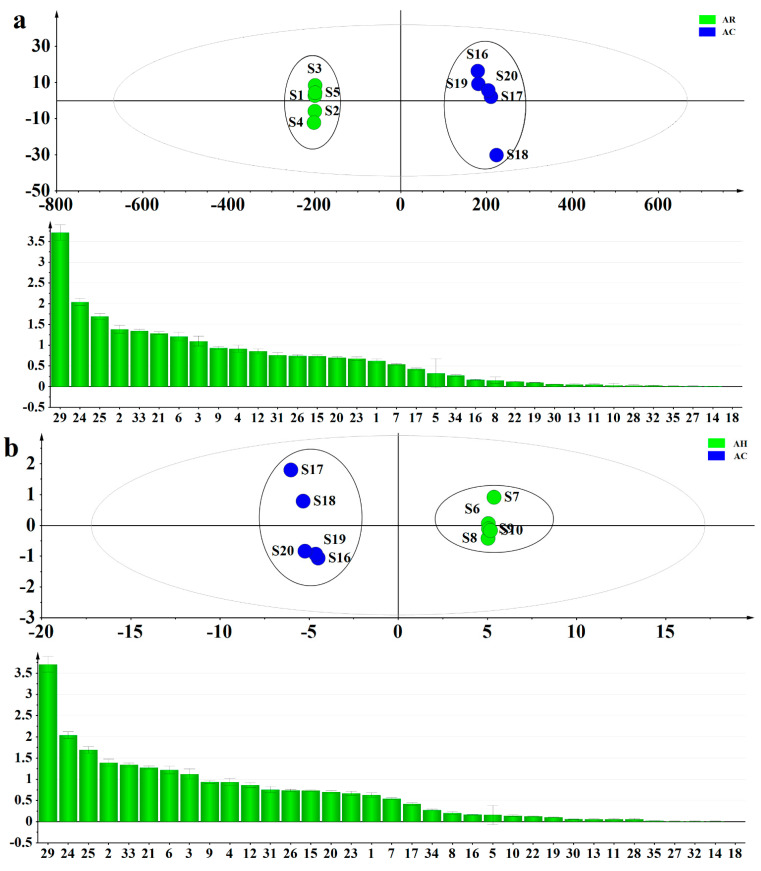

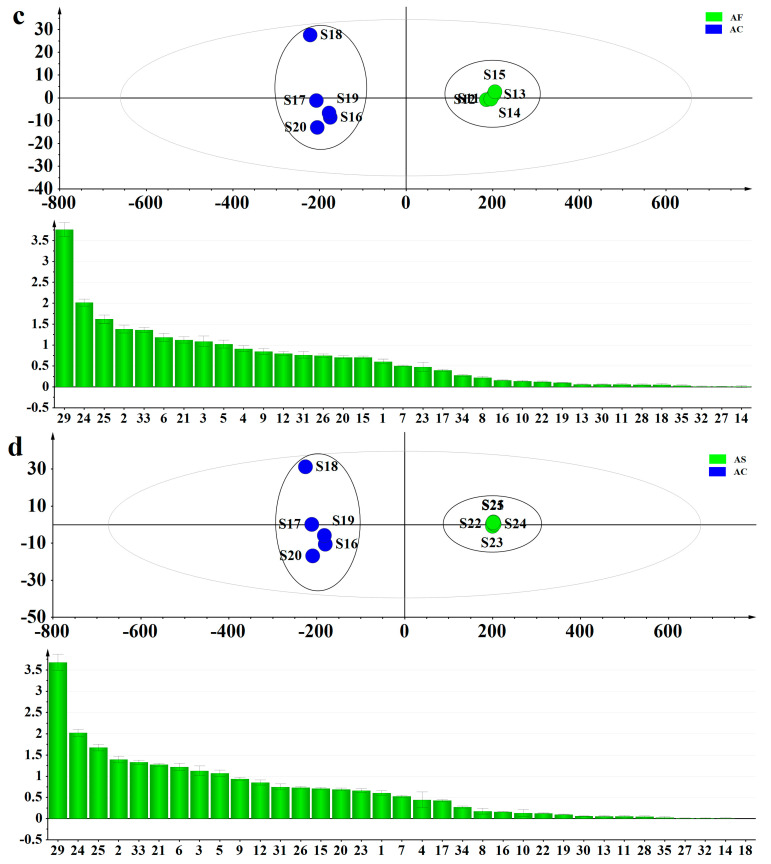
The partial least squares discriminant analysis (PLS-DA) score scatter plot and variable importance in the projection (VIP) of Abelmoschi Radix (AR) and Abelmoschi Corolla (AC) (**a**), Abelmoschi Herba (AH) and AC (**b**), Abelmoschi Folium (AF) and AC (**c**), Abelmoschi Semen (AS) and AC (**d**).

PLS-DA, a supervised pattern recognition method, was performed to differentiate AC and other parts of AM (AR, AH, AF, AS), and to find out the important constituents that cause the differences with variable importance in the projection (VIP) values. The PLS-DA score scatter plot and VIP values are shown in Figure 4a–d. The established PLS-DA model showed good adaptability (R^2^X = 0.991, 0.906, 0.995 and 0.995, R^2^Y = 0.998, 0.997, 0.997, and 0.998) and predictability (Q^2^ = 0.996, 0.992, 0.993, and 0.995). AR and AC, AH and AC, AF and AC, AS and AC were all separated into two clusters along the PC1 axis. The result indicated that the differences of constituents between AC and other parts of AM were remarkable. The VIP value was used to describe the contribution of each variable to the model and explore the differential constituents for the classification of AC and other parts of AM. A compound was selected as a potential chemical marker when the VIP value was greater than 1.0. Finally, three amino acids and five flavonoids including L-serine (**2**), L-threonine (**3**), L-valine (**6**), quercetin 3-O-robinobioside (**21**), hyperin (**24**), isoquercetin (**25**), hibifolin (**29**), quercetin 3′-O-glucoside (**33**) were screened out to discriminate AC and other parts. The VIP values of these constituents were all greater than 1.0 in the four sets of comparisons. Therefore, these constituents could be selected as chemical markers to distinguish AC and other parts of AM.

## 3. Discussion

In previous studies, AC was used as a medicinal material with high medicinal value [2,3,4]. Other parts of AM including AR, AH, AF and AS have also been recorded for medicinal purposes, but they have been underutilized. Therefore, in this research, we sought to establish a method for simultaneous determination of multiple constituents. Thirty eight ingredients including 2′-deoxyadenosine, thymidine, 2,4-dihydroxybenzoic acid and 35 constituents determined in this article were selected as initial options. However, 2′-deoxyadenosine, thymidine, and 2,4-dihydroxybenzoic acid only had weak MS response and low concentration in samples, so they were eliminated. The selected 35 constituents basically summarize all the chemical structural types of the bioactive constituents in AM, so their content variation can profile the distribution pattern of bioactive constituents in different parts of AM. Among the 35 target constituents, some are extremely similar in polarity, such as the isomers of hyperin, isoquercetin, and quercetin 7-O-glucoside while the content of the different constituents varied greatly. Therefore, UFLC-QTRAP-MS/MS was chosen as the analysis technology because of its remarkable superiority in selectivity, sensitivity, and analysis capability [24]. In summary, a method based on UFLC-QTRAP-MS/MS was established for the simultaneous determination of 35 constituents in the different parts of AM.

After analysis of the content in different parts of AM, we found that flavonoids and amino acids were the major components among the different parts, while flavonoids and amino acids in AM were mainly distributed in AC. The results of PCA showed that there were significant differences between AC and other parts of AM. The result of PLS-DA revealed that the metabolites between AC and other parts of AM were significantly different and eight different compounds including L-serine, L-threonine, L-valine, quercetin 3-O-robinobioside, hyperin, isoquercetin, hibifolin, and quercetin 3′-O-glucoside were picked out as the chemical markers. Therefore, we speculated that the difference in the content of flavonoids and amino acids might be an important reason for the unbalanced application in different parts of AM.

## 4. Materials and Methods

### 4.1. Plant Materials

**Figure 5:**
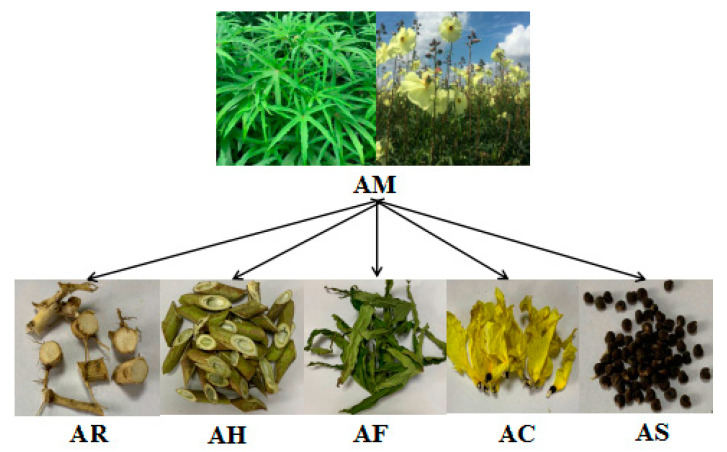
Five segments (AR, AH, AF, AC, and AS) of AM.

The AR (S1–S5), AH (S6–S10), AF (S11–S15), AC (S16–S20), and AS (S21–S25) samples were collected from Xinghua City, Jiangsu Province (32°98′17″ N, 119°90′44″ E) in the traditional harvest time and dried in an oven, as shown in Figure 5. All the samples were authenticated by Professor Xunhong Liu (Nanjing University of Chinese Medicine, Nanjing, China) and deposited in the laboratory of Chinese medicine identification, Nanjing University of Chinese Medicine.

### 4.2. Chemicals and Reagents

The standards of L-lysine (**1**), L-serine (**2**), L-threonine (**3**), L-glutamic acid (**4**), L-proline (**5**), L-valine (**6**), L-tyrosine (**7**), adenosine (**8**), L-isoleucine (**9**), guanosine (**10**), inosine (**11**), L-leucine (**12**), 5-(hydroxymethyl)-2-furancarboxylic acid (**14**), L-phenylalanine (**15**), chlorogenic acid (**17**), caffeic acid (**18**), myricetin 3′-O-glucoside (**26**), 3,4-dicaffeoylquinic acid (**27**), 3,5-dicaffeoylquinic acid (**28**), and 4,5-dicaffeoylquinic acid (**32**) were purchased from Shanghai Yuanye Biotechnology Co., Ltd. (Shanghai, China). 3,4,5-Trihydroxybenzoic acid (**13**), rutin (**23**), hyperin (**24**), and quercetin (**34**) were purchased from the Chinese National Institute for the Control of Pharmaceutical and Biological Products (Beijing, China). 3,4-Dihydroxybenzoic acid (**16**) was purchased from Shanghai Ronghe Pharmaceutical Technology Co., Ltd. (Shanghai, China). Dihydromyricetin (**19**) and myricetin (**31**) were purchased from Chengdu Aifa Bio-technology Co., Ltd. (Chengdu, China). Myricetin 3-O-glucoside (**20**) and quercetin 3-O-robinobioside (**21**) were purchased from Liangwei Bio-technology Co., Ltd. (Nanjing, China). Quercetin 7-O-glucoside (**22**), hibifolin (**29**), and quercetin 3-O-(6-acetylglucoside) (**30**) were purchased from Nanjing Casses Pharmaceutical Technology Co., Ltd. (Nanjing, China). Isoquercetin (**25**), quercetin 3′-O-glucoside (**33**), and tiliroside (**35**) were purchased from Chengdu Chroma-Biotechnology Co., Ltd. (Chengdu, China). The purities of **14**, **26**, and **32** were above 97% and other standards were greater than 98%, tested by HPLC analysis. The structures of the 35 standards are shown in Figure S1. Formic acid, acetonitrile and methanol of HPLC grade were purchased from Merck (Darmstadt, Germany). The deionized water was prepared by a Milli-Q water purification system (Millipore, Bedford, MA, USA).

### 4.3. Preparation of Standard Solutions

Each reference compound was accurately weighed and completely dissolved in 70% (*v*/*v*) methanol to produce their respective stock solutions. A standard solution containing the 35 components was then diluted with 70% (*v*/*v*) methanol to obtain a series of standard working solutions that were used to construct calibration curves. All of the solutions were stored at 4 °C and then filtered through 0.22 µm membranes (Jinteng laboratory equipment Co., Ltd., Tianjin, China) before LC-MS analysis.

### 4.4. Preparation of Sample Solutions

Extraction variables, including extraction solvent (50% methanol, 60% methanol, 70% methanol, 80% methanol, 90% methanol, and 100% methanol), solid–liquid ratio (1:10, 1:20, 1:30, 1:40, and 1:50 g/mL) and ultrasonic extraction time (15, 30, 45, 60, 75, and 90 min) were optimized in order to obtain a suitable extraction condition. Then the AM was divided into five parts as AR, AH, AF, AC, AS. All samples were crushed into powder and screened through a 50-mesh sieve. The sample powder (0.5 g) was accurately weighed and then ultrasonically extracted with 20 mL 70% (*v*/*v*) methanol for 30 min, respectively. After cooling to room temperature, the same solvent was added to compensate for the weight lost during extraction. Then the extract was filtered, and the filtrate was centrifuged at 12,000 r/min for 10 min. Afterwards, the supernatant was diluted 20 times and filtered through a 0.22 µm membrane before LC-MS analysis.

### 4.5. Chromatographic and Mass Spectrometric Conditions

The chromatographic analysis was performed using a SHIMADZU UFLC XR system (Shimadzu Co., Kyoto, Japan), which consisted of an LC-20AD binary pump, a SIL-20A XR auto sampler, and a CTO-20AC column oven.

The key factors affecting chromatographic separation were fully optimized. Three types of columns: Synergi^TM^ Hydro-RP 100 Å column (2.0 mm × 100 mm, 2.5 µm), ZORBAX Extend-C_18_ (2.1 mm × 100 mm, 1.8 μm) and XBridge^®^C_18_ column (4.6 mm × 100 mm, 3.5 µm) were investigated for the separation effect of the 35 target constituents. In addition, different kinds of mobile phases (water–methanol, water–acetonitrile, 0.1% (*v*/*v*) aqueous formic acid water solution–acetonitrile, 0.1% (*v*/*v*) aqueous formic acid water solution–methanol:acetonitrile (1:1)), flow rates (0.5, 0.6, and 0.7 mL/min) and column temperatures (25, 30, 35 °C) were examined.

The mass spectrometric detection was performed on an API5500 triple quadrupole/linear ion trap mass spectrometer (AB Sciex, Framingham, MA, USA), which was equipped with an electrospray ionization (ESI) source operating under both positive and negative ion modes. The operation parameters of the mass spectrometer were set as follows: the ion source temperature (TEM), 550 °C; the spray voltage (IS), 4500 V (positive mode), –4500 V (negative mode); the flow rate of curtain gas (GUR), 40 L/min.; the flow rate of nebulization gas (GS1), 55 L/min; the flow rate of auxiliary gas (GS2), 55 L/min.

The standard solution of each target constituent with a mass concentration of 100 ng/mL was injected into the electrospray ionization (ESI) source, and a full scan was performed in the positive and negative ion modes.

### 4.6. Validation of the Method

Validation of the method was carried out on the basis of the International Conference on Harmonization (ICH) guidelines Q2 (R1) [25], in terms of linearity and range, limits of detection (LOD) and limits of quantification (LOQ), precision, repeatability, stability, accuracy.

A series of standard working solutions containing the 35 compounds was analyzed from low to high concentrations to establish calibration curves. Plotting the peak area (*Y*) versus the corresponding concentration (*X*) constructed the calibration curves. Subsequently, the regression equation, correlation coefficient, and linear range were calculated; the LOD and LOQ of each analyte were measured at signal-to-noise ratio (S/N) of about 3 and 10, respectively.

Intra-day and inter-day precision were determined with the standard solution 6 times within a single day and 3 times within three consecutive days. The relative standard deviation (RSD) of the peak area was taken as a measure of precision. The same sample was divided into 6 parts in parallel, and the samples were extracted and analyzed by the above method. The RSD of the peak area was taken as a measure of repeatability. The same sample was analyzed at 0 h, 2 h, 4 h, 8 h, 12 h, and 24 h to evaluate the inherent stability characteristics of each compound. The RSD of the peak area was taken as a measure of stability.

A standard addition method for a recovery test was performed to evaluate the accuracy of the established method. The test was carried out by adding certain amounts of standard (approximately equivalent to 80%, 100%, 120% levels of each compound) to the sample which had known content of the 35 ingredients. Each level of addition was repeated three times and the spiked samples were extracted and analyzed using the above mentioned method. The extraction recovery rate of each compound was calculated by the following formula: recovery(%) = (measured amount − original amount in sample)/spiked amount × 100%.

### 4.7. Multivariate Statistical Analysis

In order to get a good overview of the sample classification from the different parts of AM, the data of 35 components were used to carry out PCA, which is an unsupervised pattern recognition method, with the software of SIMCA-P 13.0 (Umetrics AB, Umea, Sweden). PLS-DA, was applied to disclose which chemical components contributed most to the clusters of AC and other parts of AM, and VIP maps were obtained in the model. The histograms were charted by GraphPad Prism 8.0 software (Graphpad Software, San Diego, CA, USA).

## 5. Conclusions

In this study, a reliable analytical method based on UFLC-QTRAP-MS/MS was established for the simultaneous determination of 14 flavonoids, eight organic acids, three nucleosides and 10 amino acids in the different parts of AM. Furthermore, the contents of 35 constituents in the different parts of AM were compared and evaluated combined with multivariate statistical analysis. The result proved that the contents of flavonoids and amino acids account for a high proportion in the different parts of AM, and the contents of nucleosides and organic acids in each part of AM were at a relatively low level. The results of PCA showed a significant difference between AC and other parts of AM. The result of PLS-DA showed that the metabolites between AC and other parts of AM were significantly different and eight different compounds (L-serine, L-threonine, L-valine, quercetin 3-O-robinobioside, hyperin, isoquercetin, hibifolin, and quercetin 3′-O-glucoside) were significantly related to the sample classification. The research does not just provide the basic information for revealing the distribution patterns in AC and other parts of AM from the same origin, but also complements some of the scientific data for quality comprehensive evaluation of AC.

## Figures and Tables

**Table 1:** Retention time, related mass spectrometric data of the 35 target constituents.

No.	Compounds	*t_R_* (min)	MRM Transitions (*m/z*)	DP(V)	CE(eV)	Ion Mode
1	L-Lysine	1.77	147.1/83.9	100	14	ESI^+^
2	L-Serine	1.90	106.1/60.0	100	8	ESI^+^
3	L-Threonine	1.97	120.1/74.0	100	20	ESI^+^
4	L-Glutamic acid	1.99	148.1/83.9	12	14	ESI^+^
5	L-Proline	2.13	116.1/70.0	68	10	ESI^+^
6	L-Valine	2.66	118.1/72.1	100	10	ESI^+^
7	L-Tyrosine	4.02	182.1/136.0	16	16	ESI^+^
8	Adenosine	4.17	268.1/136.1	86	23	ESI^+^
9	L-Isoleucine	4.33	132.1/86.1	100	16	ESI^+^
10	Guanosine	4.40	284.3/152.1	42	16	ESI^+^
11	Inosine	4.41	269.0/137.0	46	15	ESI^+^
12	L-Leucine	4.60	132.2/86.0	64	10	ESI^+^
13	3,4,5-Trihydroxybenzoic acid	4.70	169.0/125.0	−33	−13	ESI^−^
14	5-(Hydroxymethyl)-2-furancarboxylic acid	5.92	141.0/97.0	−35	−12	ESI^−^
15	L-Phenylalanine	5.94	166.1/120.1	100	14	ESI^+^
16	3,4-Dihydroxybenzoic acid	6.84	152.9/109.0	−85	−16	ESI^−^
17	Chlorogenic acid	8.30	353.1/190.9	−35	−20	ESI^−^
18	Caffeic acid	9.87	174.0/134.9	−125	−20	ESI^−^
19	Dihydromyricetin	10.48	319.0/193.0	−44	−10	ESI^−^
20	Myricetin 3-O-glucoside	12.71	479.0/316.0	−155	−36	ESI^−^
21	Quercetin 3-O-robinobioside	15.92	609.0/299.9	−170	−48	ESI^−^
22	Quercetin 7-O-glucoside	15.93	463.1/301.0	−38	−28	ESI^−^
23	Rutin	16.33	609.0/299.9	−170	−48	ESI^−^
24	Hyperin	16.58	462.9/300.0	−155	−36	ESI^−^
25	Isoquercetin	17.25	462.9/300.0	−155	−36	ESI^−^
26	Myricetin 3′-O-glucoside	17.80	479.0/317.0	−90	−36	ESI^−^
27	3,4-Dicaffeoylquinic acid	19.04	515.0/353.0	−80	−26	ESI^−^
28	3,5-Dicaffeoylquinic acid	19.35	515.0/353.0	−75	−24	ESI^−^
29	Hibifolin	22.40	493.2/317.0	−155	−30	ESI^−^
30	Quercetin 3-O-(6-acetylglucoside)	22.80	505.0/300.0	−75	−38	ESI^−^
31	Myricetin	23.03	317.2/179.0	−24	−24	ESI^−^
32	4,5-Dicaffeoylquinic acid	24.36	515.0/353.0	−75	−24	ESI^−^
33	Quercetin 3′-O-glucoside	27.50	463.1/301.0	−38	−28	ESI^−^
34	Quercetin	29.81	301.1/151.0	−62	−28	ESI^−^
35	Tiliroside	29.89	593.0/284.9	−175	−38	ESI^−^

**Table 2:** Regression equation, limits of detections (LODs), limits of quantifications (LOQs), precision, repeatability, stability, and recovery of the 35 investigated constituents.

No.	Compounds	Regression Equation	*r*	Linear Range (ng/mL)	LOD (ng/mL)	LOQ (ng/mL)	Precision(RSD,%)		Repeatability (RSD, %) (*n* = 6)	Stability (RSD, %) (*n* = 6)	Recovery(%)	
							Intra-Day (*n* = 6)	Inter-Day (*n* = 9)			Mean	RSD
1	L-Lysine	*Y* = 1310*X* − 35,500	0.9994	50.30–2515	7.86	26.20	2.7	4.9	4.9	2.8	104.4	4.2
2	L-Serine	*Y* = 788*X* − 27,600	0.9991	51.20–10,240	9.85	32.82	2.8	4.1	2.0	2.4	101.1	1.3
3	L-Threonine	*Y* = 853*X* − 17,100	0.9992	51.60–5160	12.48	41.61	4.8	4.3	3.3	3.0	100.3	4.0
4	L-Glutamic acid	*Y* = 2470*X* − 116,000	0.9998	51.40–5140	10.42	34.73	4.3	4.3	4.8	3.8	101.1	3.7
5	L-Proline	*Y* = 3720*X* + 66,700	0.9999	25.20–10,080	2.04	6.81	3.7	4.8	2.9	3.4	100.3	1.1
6	L-Valine	*Y* = 8050*X* − 312,000	0.9996	49.90–9980	14.39	47.98	3.8	4.9	2.7	2.1	104.4	2.3
7	L-Tyrosine	*Y* = 4610*X* + 22,500	0.9994	12.43–4970	1.55	5.18	2.9	4.1	3.6	4.7	99.04	4.6
8	Adenosine	*Y* = 18,100*X* + 212,000	0.9997	1.00–2500	0.22	0.74	3.3	4.9	3.6	3.2	99.64	0.22
9	L-Isoleucine	*Y* = 14,100*X* + 199,000	0.9990	25.00–5000	5.07	16.89	2.8	4.9	3.2	3.3	101.7	4.4
10	Guanosine	*Y* = 7050*X* + 94,000	0.9994	1.03–2560	0.15	0.51	2.6	4.5	4.6	4.5	100.2	0.38
11	Inosine	*Y* = 6560*X* + 99,300	0.9994	4.96–1240	1.24	4.13	2.9	4.0	3.2	3.4	100.0	0.44
12	L-Leucine	*Y* = 8810*X* + 397,000	0.9993	12.63–2520	1.84	6.13	1.1	4.2	2.9	3.8	98.43	4.3
13	3,4,5-Trihydroxybenzoic acid	*Y* = 4330*X* + 49,300	0.9997	13.31–532	3.73	12.44	4.7	4.3	4.9	4.9	99.43	2.1
14	5-(Hydroxymethyl)-2-furancarboxylic acid	*Y* = 2400*X* − 20,200	0.9992	5.07–5070	1.36	4.53	4.6	4.9	4.9	4.3	103.3	2.6
15	L-Phenylalanine	*Y* = 17,900*X* + 779,000	0.9997	0.50–2480	0.14	0.46	3.9	4.8	1.9	4.9	99.53	3.2
16	3,4-Dihydroxybenzoic acid	*Y* = 12,400*X* + 78,100	0.9996	5.21–261	1.09	3.62	3.7	4.3	3.4	2.8	99.46	1.7
17	Chlorogenic acid	*Y* = 6400*X* + 97,400	0.9994	2.48–2480	0.41	1.38	4.6	4.3	2.1	4.9	100.4	4.3
18	Caffeic acid	*Y* = 17,400*X* + 200,000	0.9990	2.48–1238	0.73	2.43	3.6	4.8	4.6	4.3	99.41	2.0
19	Dihydromyricetin	*Y* = 5250*X* + 68,800	0.9991	2.54–1268	0.51	1.71	4.4	4.8	3.4	4.5	100.8	4.9
20	Myricetin 3-O-glucoside	*Y* = 4650*X* + 155,000	0.9999	5.03–10,050	0.37	1.23	4.7	4.8	2.5	4.2	100.0	4.9
21	Quercetin 3-O-robinobioside	*Y* = 2630*X* − 20,500	0.9999	5.03–10,100	0.70	2.34	2.5	4.2	1.0	4.2	101.5	2.8
22	Quercetin 7-O-glucoside	*Y* = 7500*X* + 79,400	0.9992	2.55–1273	0.37	1.24	4.2	4.5	2.9	4.8	103.3	4.2
23	Rutin	*Y* = 3110*X* − 33,100	0.9999	2.51–5020	0.22	0.72	3.5	4.9	2.3	4.4	100.9	1.0
24	Hyperin	*Y* = 5790*X* + 318,000	0.9997	5.05–20,200	0.20	0.67	4.5	4.9	1.1	4.7	99.84	1.2
25	Isoquercetin	*Y* = 5230*X* − 6260	0.9999	5.05–20,200	0.92	3.06	4.6	4.2	1.0	4.5	100.4	1.2
26	Myricetin 3′-O-glucoside	*Y* = 6460*X* − 104,000	0.9997	5.25–10.500	1.23	4.10	2.7	4.9	1.8	4.2	100.2	1.4
27	3,4-Dicaffeoylquinic acid	*Y* = 3300*X* − 153,000	0.9998	12.63–1263	3.01	10.03	4.9	4.8	3.8	3.9	101.7	1.2
28	3,5-Dicaffeoylquinic acid	*Y* = 2760*X* − 19,000	0.9993	5.02–1255	1.47	4.90	2.4	3.6	3.2	4.8	98.06	1.8
29	Hibifolin	*Y* = 1620*X* − 1320,000	0.9991	253.00–50,600	66.00	220.00	4.2	4.8	4.6	4.4	100.6	1.2
30	Quercetin 3-O-(6-acetylglucoside)	*Y* = 7120*X* + 122,000	0.9998	2.56–5125	0.17	0.56	4.6	4.8	3.8	4.3	99.41	2.1
31	Myricetin	*Y* = 4150*X* − 22,300	0.9990	5.01–5010	1.25	4.18	4.7	4.9	3.4	2.8	101.9	2.6
32	4,5-Dicaffeoylquinic acid	*Y* = 4410*X* − 101,000	0.9998	12.43–2490	1.61	5.36	4.5	4.8	4.7	4.3	101.0	2.7
33	Quercetin 3′-O-glucoside	*Y* = 7930*X* + 503,000	0.9993	5.00–10,000	0.32	1.07	3.9	4.4	1.8	4.2	99.86	1.3
34	Quercetin	*Y* = 9450*X* − 17,300	1.0000	0.51–2-560	0.08	0.27	3.9	4.7	4.9	3.8	102.1	1.6
35	Tiliroside	*Y* = 9040*X* + 18,100	0.9999	0.51–256	0.07	0.22	4.1	4.4	3.7	4.0	101.0	3.2

## Data Availability

The data presented in this study are available in this article or in supplementary material.

## Supplementary Materials

The following are available online, Figure S1: Chemical structures of 35 constituents, Table S1: Contents of 35 constituents in AR, AH, and AF, Table S2: Contents of 35 constituents in AC and AS.
