# Supplementary material for: Comparison of Multiple Bioactive Constituents in the Corolla and Other Parts of Abelmoschus manihot
Source: Molecules. 2021 Mar 25;26(7):1864. doi: 10.3390/molecules26071864 (PMC8037085; doi:10.3390/molecules26071864)
Supplement: Supplementary file 1 [file molecules-26-01864-s001.pdf]

Supplementary Material

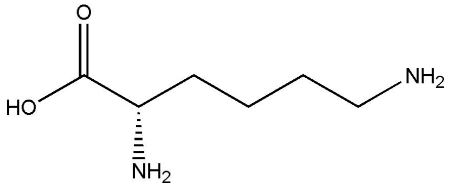

L-Lysine (1)

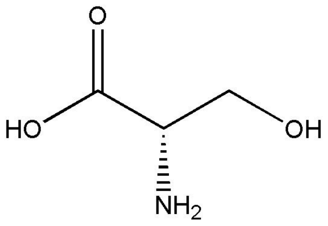

L-Serine (2)

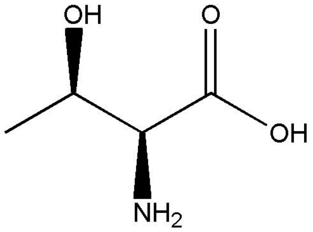

L-Threonine (3)

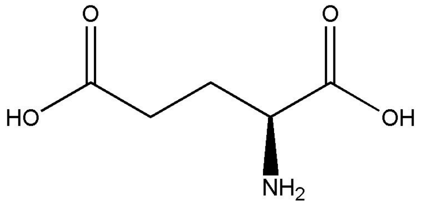

L-Glutamic acid (4)

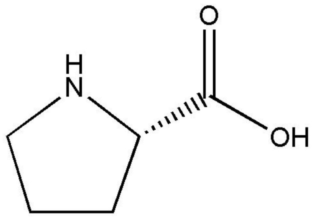

L-Proline (5)

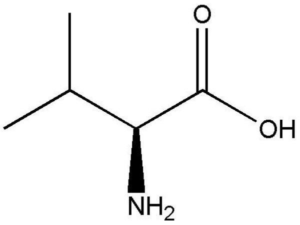

L-Valine (6)

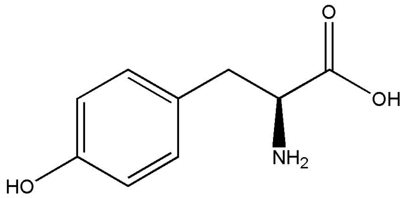

L-Tyrosine (7)

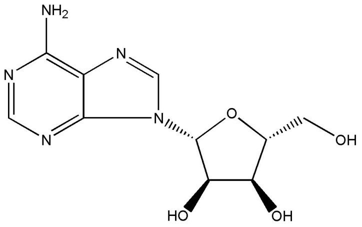

Adenosine (8)

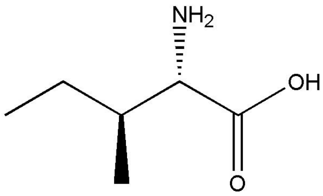

L-Isoleucine (9)

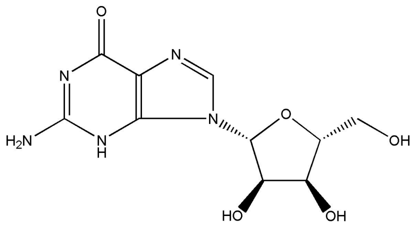

Guanosine (10)

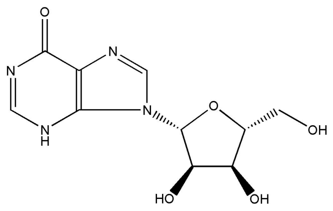

Inosine (11)

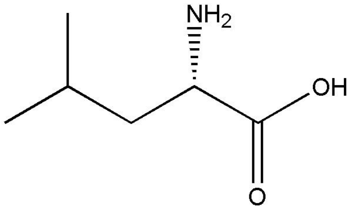

L-Leucine (12)

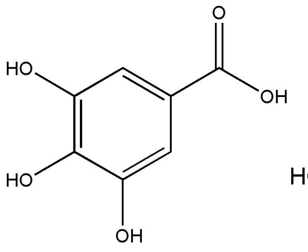

3,4,5-Trihydroxybenzoic acid (13)

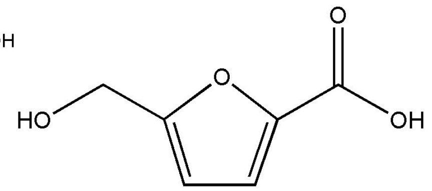

5-(Hydroxymethyl)-2-furancarboxylic acid (14)

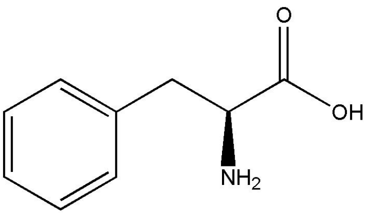

L-Phenylalanine (15)

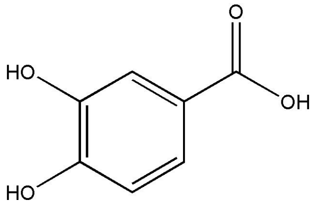

3,4-Dihydroxybenzoic acid (16)

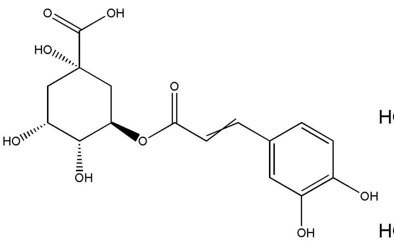

Chlorogenic acid (17)

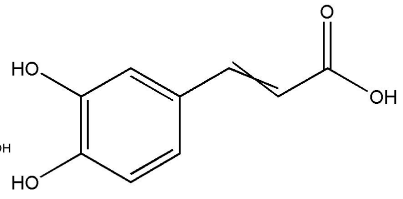

Caffeic acid (18)

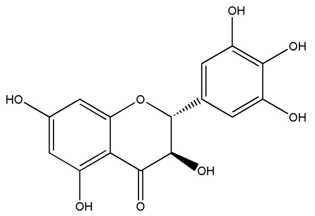

Dihydromyricetin (19)

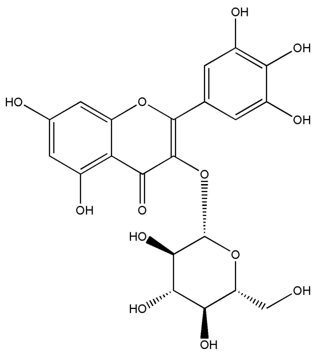

Myricetin 3-O-glucoside (20)

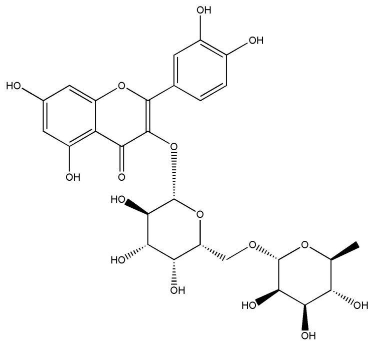

Quercetin 3-O-robinobioside (21)

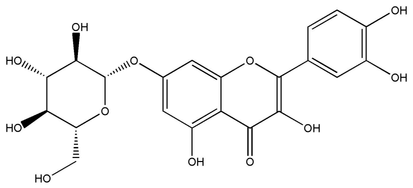

Quercetin 7-O-glucoside (22)

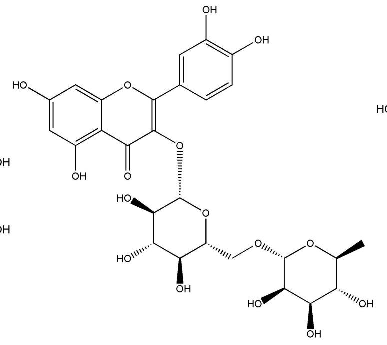

Rutin (23)

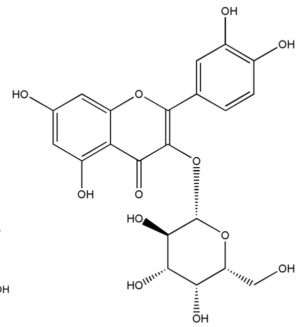

Hyperin (24)

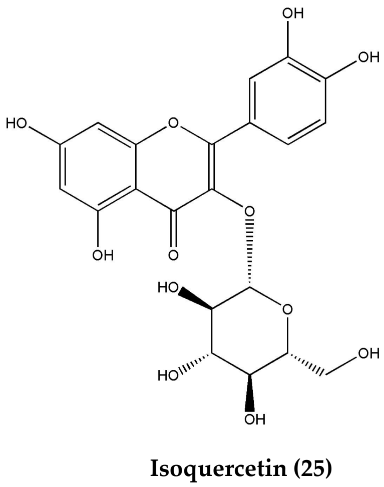

Isoquercetin (25)

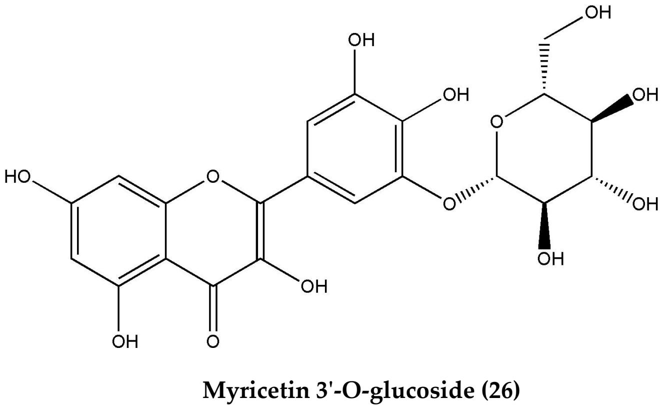

Myricetin 3'-O-glucoside (26)

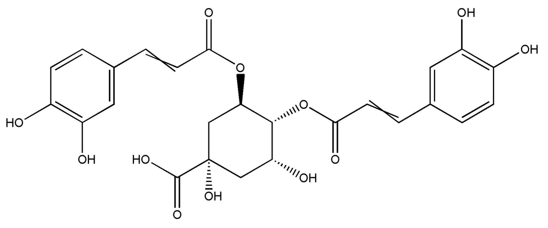

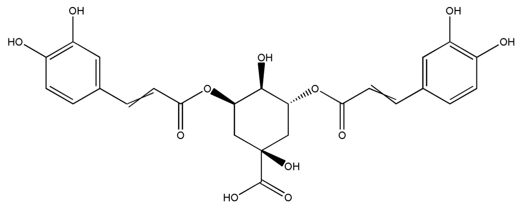

3,4-Dicaffeoylquinic acid (27) **3,5-Dicaffeoylquinic acid (28)**

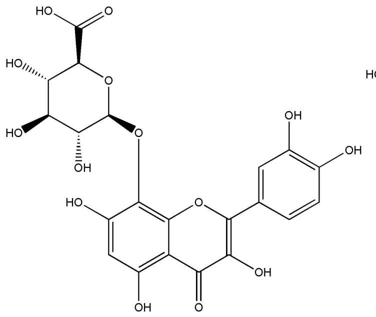

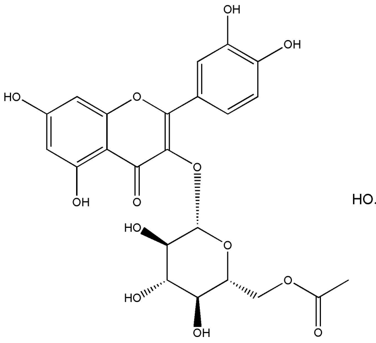

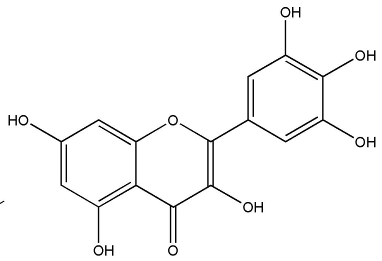

Hibifolin (29) **Quercetin 3-O-(6-acetylglucoside) (30)** **Myricetin (31)**

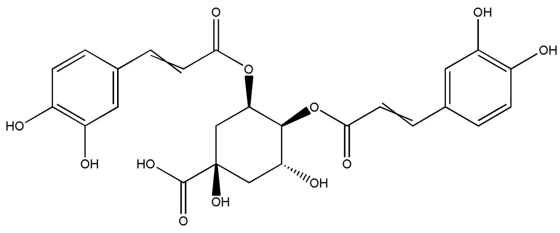

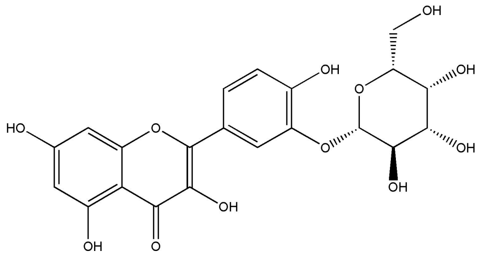

4,5-Dicaffeoylquinic acid (32) **Quercetin 3'-O-glucoside (33)**

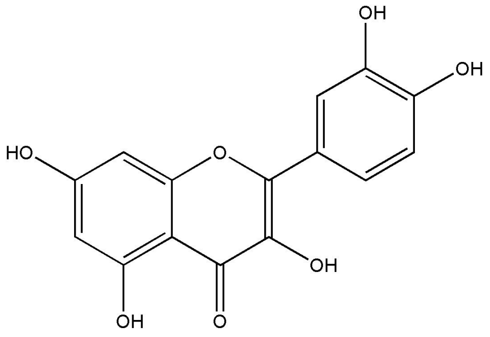

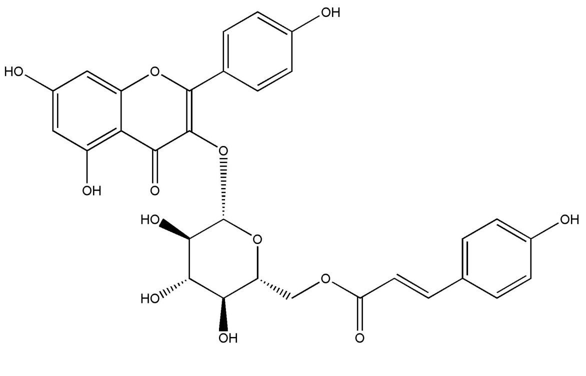

Quercetin (34) **Tiliroside (35)**

Figure S1. Chemical structures of 35 constituents.

Table S1. Contents of 35 constituents in AR, AH and AF. (μg/g, n = 3).

No.	Compounds	AR					AH					AF				
		S1	S2	S3	S4	S5	S6	S7	S8	S9	S10	S11	S12	S13	S14	S15
1	L-Lysine	57.37	66.13	68.74	59.94	62.79	45.63	44.14	46.14	45.84	45.42	130.36	141.29	99.89	132.93	82.62
2	L-Serine	339.49	298.31	318.25	313.49	327.58	271.40	218.84	280.64	245.18	245.52	486.81	507.92	304.28	653.92	290.73
3	L-Threonine	335.65	445.94	404.15	255.35	400.64	205.28	161.37	226.92	203.53	256.39	544.89	639.34	320.03	536.98	323.40
4	L-Glutamic acid	361.24	374.71	244.11	337.04	334.32	231.67	231.21	268.66	228.57	267.47	384.05	500.43	309.44	436.30	275.73
5	L-Proline	3317.00	3622.96	3016.71	3815.90	3212.10	2499.29	3404.33	2844.16	2887.73	4700.03	568.34	823.87	306.21	678.67	288.65
6	L-Valine	245.52	242.88	214.77	184.16	232.79	161.03	128.07	179.97	151.19	151.41	500.97	611.01	273.60	490.53	269.30
7	L-Tyrosine	19.34	49.58	45.36	28.56	27.68	36.49	22.64	34.95	25.59	38.65	137.33	141.26	145.22	199.29	91.99
8	Adenosine	59.99	123.33	110.36	119.32	98.49	63.94	63.55	63.52	62.21	68.05	30.18	56.89	59.60	62.28	38.78
9	L-Isoleucine	156.56	113.62	109.52	95.43	126.61	86.21	51.68	80.58	59.61	63.10	635.39	748.54	325.86	530.98	299.39
10	Guanosine	29.03	57.35	61.14	55.18	51.76	7.26	10.66	9.87	7.60	6.47	10.89	9.53	11.35	12.72	7.82
11	Inosine	-	-	-	-	-	-	-	-	-	-	-	-	-	-	-
12	L-Leucine	49.55	45.08	54.73	53.97	47.05	38.37	13.62	40.38	18.06	19.82	342.54	375.98	206.48	357.43	218.03
13	3,4,5-Trihydroxybenzoic acid	-	-	-	-	-	-	-	-	-	-	-	-	-	-	-
14	5-(Hydroxymethyl)-2-furancarboxylic acid	8.41	8.12	7.04	6.89	7.39	7.50	9.20	7.29	7.51	7.54	7.01	7.95	7.34	7.45	8.34
15	L-Phenylalanine	33.54	28.22	27.74	37.16	25.08	28.17	17.02	37.56	20.60	26.89	129.63	239.90	97.51	183.88	107.67
16	3,4-Dihydroxybenzoic acid	-	-	-	-	-	-	-	-	-	-	5.54	5.67	8.71	7.29	4.51
17	Chlorogenic acid	-	-	-	-	-	36.04	27.07	-	10.82	3.58	88.68	74.41	77.98	54.74	44.29
18	Caffeic acid	-	-	-	-	-	-	-	-	-	-	6.62	11.58	3.36	5.57	5.05
19	Dihydromyricetin	-	-	-	-	-	-	-	-	-	-	-	-	-	-	-
20	Myricetin 3-O-glucoside	28.83	28.13	28.16	28.01	27.13	28.55	30.94	27.49	27.34	27.18	27.53	27.69	27.26	27.27	27.15

21	Quercetin 3-O-robinobioside	8.40	7.69	7.50	7.99	7.07	95.87	70.40	29.49	36.52	14.22	964.22	1389.43	1059.13	812.97	842.06
22	Quercetin 7-O-glucoside	-	-	-	-	-	-	-	-	-	-	-	-	-	-	-
23	Rutin	10.54	10.02	11.16	10.57	10.64	42.68	31.74	19.90	19.10	11.36	538.31	748.90	512.90	417.85	471.16
24	Hyperin	-	-	-	-	-	62.53	50.97	-	-	-	518.31	711.42	488.21	426.18	355.09
25	Isoquercetin	10.88	6.04	5.59	6.16	4.32	41.15	46.53	11.67	11.75	5.49	881.85	1043.54	590.10	617.90	583.28
26	Myricetin 3'-O-glucoside	16.42	13.88	14.32	14.18	13.52	14.69	13.58	13.16	13.10	13.15	13.19	12.97	12.93	12.99	13.17
27	3,4-Dicaffeoylquinic acid	38.26	37.63	37.59	37.52	37.70	37.29	38.08	37.46	37.38	37.58	38.31	37.21	37.66	37.30	37.36
28	3,5-Dicaffeoylquinic acid	8.03	6.76	6.57	6.49	8.02	17.49	14.63	13.62	14.52	13.46	13.45	13.76	14.03	11.49	13.70
29	Hibifolin	656.44	654.69	654.79	656.68	656.11	665.51	675.82	654.02	658.01	654.61	652.85	654.13	653.50	654.57	653.06
30	Quercetin 3-O-(6-acetylglucoside)	-	-	-	-	-	-	-	-	-	-	-	-	-	-	-
31	Myricetin	5.25	4.65	5.19	4.97	4.78	4.83	4.81	4.98	4.97	4.58	4.57	5.02	5.06	4.71	4.61
32	4,5-Dicaffeoylquinic acid	22.46	20.86	20.80	20.10	19.24	18.61	18.79	18.58	18.63	18.76	18.58	18.59	18.77	18.54	18.50
33	Quercetin 3'-O-glucoside	-	-	-	-	-	-	-	-	-	-	-	-	-	-	-
34	Quercetin	11.62	8.68	6.63	5.29	4.72	2.91	3.04	2.22	2.16	1.77	5.20	4.81	2.96	2.30	3.23
35	Tiliroside	-	-	-	-	-	-	-	-	-	-	6.18	3.06	2.22	3.09	4.30

Note: “-”not detected.

Table S2. Contents of 35 constituents in AC and AS. (μg/g, n = 3).

No.	Compounds	AC					AS				
		S16	S17	S18	S19	S20	S21	S22	S23	S24	S25
1	L-Lysine	894.25	936.77	969.02	1005.49	1150.30	77.86	126.23	113.26	49.74	102.19
2	L-Serine	4704.46	4774.51	4854.24	4772.00	5525.03	139.81	208.94	165.04	139.61	128.30
3	L-Threonine	3342.79	3004.83	3066.56	3075.33	3866.80	102.31	159.69	152.00	80.98	131.26
4	L-Glutamic acid	2134.65	2603.39	2176.96	2468.20	2305.71	1749.09	1982.87	1883.35	1662.48	1678.00
5	L-Proline	2757.64	2907.48	3084.90	2912.13	3466.06	131.78	211.67	280.22	83.67	209.10
6	L-Valine	3585.90	3475.98	3800.51	3531.26	4319.85	76.68	95.20	83.96	61.18	89.45
7	L-Tyrosine	691.42	783.16	732.47	739.27	769.30	72.16	81.73	65.50	41.71	67.87
8	Adenosine	151.83	195.08	184.37	155.59	148.71	96.78	112.29	66.20	45.85	106.77
9	L-Isoleucine	2160.03	2182.27	2197.56	2157.38	2272.96	32.99	45.49	31.54	25.97	37.41
10	Guanosine	47.28	68.92	58.26	60.41	49.99	130.15	167.66	92.80	67.14	97.04
11	Inosine	5.20	13.00	7.53	7.41	7.88	-	-	-	-	-
12	L-Leucine	1569.96	1996.01	1845.11	1881.08	1723.86	11.09	25.18	19.07	5.72	13.97
13	3,4,5-Trihydroxybenzoic acid	6.02	11.02	10.85	3.22	6.94	-	-	-	-	-
14	5-(Hydroxymethyl)-2-furancarboxylic acid	7.40	8.75	7.57	7.11	7.48	7.14	7.45	7.34	7.28	9.60
15	L-Phenylalanine	1273.66	1362.70	1329.38	1338.05	1304.66	110.52	108.76	83.60	72.40	102.29
16	3,4-Dihydroxybenzoic acid	58.53	67.80	70.50	56.54	72.95	-	-	-	-	-
17	Chlorogenic acid	433.73	474.85	411.90	425.58	457.37	-	-	-	-	-
18	Caffeic acid	-	-	-	-	-	-	-	-	-	-
19	Dihydromyricetin	22.41	26.52	23.06	23.05	25.00	-	-	-	-	-
20	Myricetin 3-O-glucoside	1193.88	1262.39	1173.41	1119.81	1228.02	27.70	27.13	27.07	27.09	27.18
21	Quercetin 3-O-robinobioside	3866.26	4199.75	4024.66	3871.68	3956.65	13.61	22.96	34.00	10.28	15.19
22	Quercetin 7-O-glucoside	34.06	45.14	37.53	34.00	39.81	-	-	-	-	-

23	Rutin	1154.86	1200.88	1079.47	996.89	1098.09	16.66	23.23	50.95	14.83	28.94
24	Hyperin	9854.91	10,308.45	9815.38	9647.52	10,405.07	-	-	-	-	-
25	Isoquercetin	6863.54	7534.53	6981.69	6260.93	6984.41	16.16	20.25	71.15	9.00	37.63
26	Myricetin 3'-O-glucoside	1199.54	1435.59	1363.82	1301.59	1311.88	14.69	13.23	13.27	13.60	13.51
27	3,4-Dicaffeoylquinic acid	37.29	37.23	37.21	37.20	37.24	37.40	37.51	37.44	37.42	37.72
28	3,5-Dicaffeoylquinic acid	6.14	5.94	12.76	6.22	6.03	12.53	11.69	12.49	11.47	12.25
29	Hibifolin	31,638.89	34,296.57	39,300.58	31,782.03	32,915.23	655.88	653.56	653.79	652.49	653.06
30	Quercetin 3-O-(6-acetylglucoside)	7.30	6.85	9.00	7.65	11.00	-	-	-	-	-
31	Myricetin	1117.62	1637.36	1447.38	1375.73	1350.42	4.47	4.55	4.50	4.64	4.54
32	4,5-Dicaffeoylquinic acid	18.62	18.86	19.33	18.96	18.58	18.77	18.52	18.49	18.54	18.74
33	Quercetin 3'-O-glucoside	4041.86	4645.75	4411.80	4320.07	4232.54	-	-	-	-	-
34	Quercetin	199.40	180.75	182.77	164.10	202.74	2.20	2.45	2.73	2.49	2.41
35	Tiliroside	1.08	0.95	0.88	0.50	0.40	1.74	8.32	3.85	0.52	2.30

Note: “-”not detected.
